# Editorial for the Special Issue “Metabolic Analysis of Plant Development and Defense Responses”

**DOI:** 10.3390/plants15121919

**Published:** 2026-06-22

**Authors:** Adnan Amin

**Affiliations:** Department of Life Sciences, Yeungnam University, Gyeongsan 38541, Republic of Korea; adnan.amin@yu.ac.kr

Various plant growth related functions including defence, growth, development, environmental perceptions are mainly linked by a central plant regulatory metabolism. Various general plant processes, including energy production, carbon allocation, biosynthesis of amino acids, redox balance, and maintenance of cellular hemostasis, are regulated by primary metabolism. The specialized metabolism on the other hand is responsible to produce a complex series of assorted compounds that are mainly involved in pathogen defense, stress tolerance and ecological interactions. In fact, there exists a complex integration among these with transcriptional regulation, hormonal signaling and environmental adaptation mechanisms.

To highlight recent developments and advances in plant metabolic research, this special issue “Metabolic Analysis of Plant Development and Defence Responses,” was established to project recent advances in plant metabolic research, particularly focusing the primary and secondary metabolic pathways involved in regulation of defence response and developmental progress in plants. The scope of this special issue was based on highlighting recent developments in research pertaining to the plant developmental stages (seed germination to fruiting and senescence), metabolic responses to biotic and abiotic stressors. With the aim to identify various regulatory networks, stress-associated biomarkers, studies discussing metabolomics, transcriptomics, proteomics, and integrated multi-omics are welcomed.

## 1. Metabolic Regulation of Plant Development

A comprehensive and precise metabolic coordination is needed for plant development. In fact, based on plant’s developmental demands (during germination, organ expansion, flowering, fruiting etc.), a redistribution of amino acids, carbon, nitrogen, lipids and secondary metabolites occurs. Here, the role of metabolomics becomes vital during identification of various biochemical signatures associated with these transitions. This consideration is important since plant metabolites are closely linked to phenotypes and imitate the integrated output of gene expression, enzyme activity and response to environmental stressors [1].

In addition, the plant’s developmental metabolism is also regulated by various hormone metabolite interactions. For instance, plant-stress response (both biotic and abiotic) is influenced by key plant hormones for instance Auxin, cytokinins, gibberellins, abscisic acid, jasmonates, ethylene, salicylic acid etc. through overlapping signaling networks. These interactions help plants coordinate growth with defense, especially under fluctuating environmental conditions [2].

## 2. Metabolic Reprogramming Under Abiotic Stress

Among all stressors, the plant metabolism is greatly affected by abiotic stress, since drought, salinity, heat, cold, etc., modify central carbon metabolism, amino acid pools, osmoprotectant accumulation, antioxidant capacity and energy balance. Here, the identification of stress-responsive metabolites relies on metabolomics that also contribute towards metabolic homeostasis and tolerance [3,4].

The published manuscript by Li et al. [5], in this special issue is a clear example of stress-associated metabolic regulation. In this research article, it has been proven that exogenous melatonin improved drought tolerance in rubber tree (*Hevea brasiliensis*) seedlings. This effect was evident by a marked decrease in chlorophyll degradation, hydrogen peroxide accumulation, malondialdehyde contents and injuries to the membrane. This was probably due to an efficient regulation of photosynthetic and antioxidant defense genes. This investigation further explains the dependency of stress tolerance on coordinated control of photosynthetic metabolism, detoxification of ROS (reactive oxygen species) and expression of defense-related genes.

## 3. Specialized Metabolites in Plant Defense

Another important element of plant defence is the presence of specialized metabolites including flavonoids, alkaloids, phenolics, saponins and terpenoids. These significantly contribute not only to limit the pathogenic attacks but also restrict herbivory and also increase antioxidant and stress signaling. A marked effect of these specialized metabolites is also reported in regulating developmental processes and ecological interactions [6].

In case of plant specialized metabolites, a clear distinction between stress related metabolites and ones related to stress tolerance/resistance is very challenging. However, recent advancements in integrated metabolomic, transcriptomic and genetic validation has made this challenge a bit addressable. Based on recent work, it is thus possible to support the understanding that specialized metabolites do participate in both development and defense regulation, thus clearly accenting their key implications in growth–defense related evolutionary constraints [7,8].

## 4. Hormonal Crosstalk and Growth–Defense Balance

Plant defense is strongly regulated by hormonal crosstalk. Salicylic acid, jasmonic acid, ethylene, abscisic acid, auxin, gibberellins, and brassinosteroids interact to determine whether resources are allocated toward growth, reproduction, or defense. Salicylic acid has a central role in plant immunity and interacts with other hormonal pathways to fine-tune immune output and growth–defense balance [9]. The Jasmonate based signaling in plants is also central to defense metabolism and stress adaptation pathways since it not only regulates specialized-metabolite biosynthesis and contribute towards metabolic trade-offs (growth vs. defense) [10]. A thoughtful consideration of these interactions is important for the improvement of crop resilience without imposing excessive penalties on growth or yield.

## 5. Multi-Omics Approaches for Metabolic Network Discovery

An increased reliance on integrated omics has been noticed these days in modern plant metabolic research since a robust identification of biochemical phenotypes, transcriptomics reveals regulatory gene expression is possible. Although, combined multi-omic methods can classify pathway hubs, stress biomarkers and candidate genes for crop improvement [11], yet such integrated frameworks need a careful experimental design. This is because the tissue specificity, developmental stages, stress intensity and biological replication etc. all affect the interpretation. Without these controls, omics datasets may remain descriptive rather than mechanistically informative.

## 6. Metabolic Engineering and Crop Resilience

Metabolic engineering offers practical opportunities to improve stress tolerance, nutritional quality, defense capacity, and yield stability. Engineering antioxidant systems, phenylpropanoid pathways, osmoprotectant accumulation, terpenoid biosynthesis, or hormone-related metabolic routes may enhance resilience under adverse conditions. Nevertheless, metabolic engineering must account for pathway trade-offs, because increasing one metabolite class may affect growth, reproduction, or defense allocation.

## 7. Future Perspectives

Future research should move beyond metabolite cataloging toward functional and predictive plant metabolic biology. Priority areas include improving metabolite annotation, identifying causal metabolic regulators, integrating omics with physiological phenotyping, developing stress-specific metabolic biomarkers, and applying genome editing or synthetic biology to validate candidate pathways.

Greater attention should also be given to underutilized crops, medicinal plants, and stress-resilient species. These plants may contain unique metabolic strategies that can support crop improvement, natural product discovery, and sustainable agriculture.

## 8. Conclusions

In plants, various key mechanism including stress adaptation, development and defense mainly rely on one central element i.e., “plant metabolism”. This mainly provides a coordinated framework for diverse metabolic functions including growth, energy allocation, stress responses, immune signaling and specialized-metabolite production in a programmed way. The investigations, encouraged in this special issue, greatly contribute towards a deep understanding of diversified metabolic networks regulating plant performance under developmental and environmental stressors. This special issue also provides evidence of improved crop resilience, productivity, and sustainability through integrated metabolomics, transcriptomics, proteomics, metabolic engineering, and physiological validation, future research can identify mechanisms. This Special Issue thus aimed to support this objective by taking together mechanistic, technological and translational advancements in plant metabolic biology.

## Data Availability

All papers cited in this editorial are available through the cited journal or publisher links.
